# Supplementary material for: Sorting at embryonic boundaries requires high heterotypic interfacial tension
Source: Nat Commun. 2017 Jul 31;8:157. doi: 10.1038/s41467-017-00146-x (PMC5537356; doi:10.1038/s41467-017-00146-x)
Supplement: Supplementary file 2 — Supplementary Software 1 [file 41467_2017_146_MOESM2_ESM.zip › PottsModel/SrcPottsModel/doc/model/Vector.html]

Vector


JavaScript is disabled on your browser.


Skip navigation links


- Overview
- Package
- Class
- Use
- Tree
- Deprecated
- Index
- Help

- Prev Class
- Next Class

- Frames
- No Frames

- All Classes

- Summary:
- Nested |
- Field |
- Constr |
- Method

- Detail:
- Field |
- Constr |
- Method


model

## Interface Vector

- All Known Implementing Classes:
  :   FiniteVector, HalfLine

  ---

    

  ```
  public interface Vector
  ```

- - ### Method Summary

    All Methods Instance Methods Abstract Methods

    | Modifier and Type | Method and Description |
    | --- | --- |
    | `double` | `getAngle()` |
    | `java.awt.Point` | `getOrigin()` |
    | `java.awt.Point` | `getTarget()` |
    | `double` | `measureAngle(java.awt.Point p)` |
    | `void` | `pointTowards(java.awt.Point p)` |
    | `void` | `rotate(java.awt.Point p, float angleRatio)` Move the vector towards a point by a percentage of the angle between them. |
    | `void` | `setOrigin(java.awt.Point p)` |

- - ### Method Detail


    - #### getAngle

      ```
      double getAngle()
      ```


    - #### getOrigin

      ```
      java.awt.Point getOrigin()
      ```


    - #### pointTowards

      ```
      void pointTowards(java.awt.Point p)
      ```


    - #### rotate

      ```
      void rotate(java.awt.Point p,
                  float angleRatio)
      ```

      Move the vector towards a point by a percentage of the angle between them.
      For example, rotating the angle by 1 will make the vector point towards p.
      Rotating the angle by 0 will do nothing. Rotating the angle by 0.5 will make the vector point
      in the middle of the old vector and the vector pointing to p.

      Parameters:
      :   `p` - The point to move the vector towards.
      :   `percentageMove` - The ratio of the rotation angle with respect to the point.


    - #### measureAngle

      ```
      double measureAngle(java.awt.Point p)
      ```


    - #### setOrigin

      ```
      void setOrigin(java.awt.Point p)
      ```


    - #### getTarget

      ```
      java.awt.Point getTarget()
      ```


Skip navigation links


- Overview
- Package
- Class
- Use
- Tree
- Deprecated
- Index
- Help

- Prev Class
- Next Class

- Frames
- No Frames

- All Classes

- Summary:
- Nested |
- Field |
- Constr |
- Method

- Detail:
- Field |
- Constr |
- Method
